# The SIRP family: from structural diversity and signaling mechanisms to implications in immune-related disease targeted therapeutics

**DOI:** 10.3389/fimmu.2026.1764114

**Published:** 2026-02-25

**Authors:** Yanmei Jin, Quiyang Huang, Jiaqi Song, Zain ul Abideen, Ruijiong Tan, Shaohua Xu, Ming Chen

**Affiliations:** 1 State Key Laboratory for Chemistry and Molecular Engineering of Medicinal Resources, Collaborative Innovation Center for Guangxi Ethnic Medicine, School of Chemistry and Pharmaceutical Sciences, Guangxi Normal University, Guilin, China; 2 Key Laboratory for Chemistry and Molecular Engineering of Medicinal Resources (Ministry of Education of China), Collaborative Innovation Center for Guangxi Ethnic Medicine, School of Chemistry and Pharmaceutical Sciences, Guangxi Normal University, Guilin, China

**Keywords:** SIRP family, CD47, immune regulation, phagocytosis, tumor immune escape, autoimmune disease

## Abstract

Signal regulatory proteins (SIRPs) are membrane receptors on immune cells that control immune homeostasis and inflammation. Although SIRP family members share homologous extracellular domains, they differ in intracellular motifs and function: SIRPα transduces inhibitory signals, SIRPβ associates with DAP12 to trigger activation, and SIRPγ primarily modulates adhesion and T cell responses. This review compares the structure, ligand interactions, and signaling mechanisms of SIRPα, SIRPβ, and SIRPγ, summarizes their roles in cancer, autoimmunity and neurodegeneration, and surveys therapeutic strategies that target the CD47–SIRPα axis. We highlight current clinical progress, common toxicities, and open questions that must be addressed to advance SIRP-targeted therapies.

## Graphical Abstract

**Figure:**
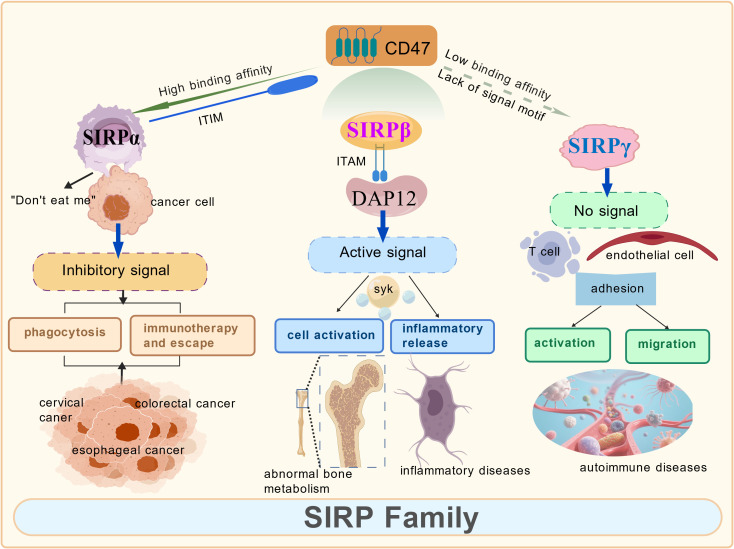
The core biological characteristics and functions of the SIRP protein family. Upon binding to CD47, SIRPα transduces an inhibitory signal that suppresses phagocytosis by macrophages. SIRPβ binds to the adaptor protein DAP12 through its transmembrane domain, thereby transmitting activation signals to promote cell activation and the release of inflammatory mediators. SIRPγ exhibits relatively low binding affinity to CD47 and lacks typical signaling motifs. Its primary function is to enhance adhesion between T cells and endothelial cells, thereby facilitating T cell activation and migration.

## Highlights

- Systematically compared the structural basis for the functional differences in SIRPα, SIRPβ, and SIRPγ signaling pathways.
- The SIRP family plays a unique role in cancer and immune-related diseases.
- Blocking the SIRPα-CD47 axis represents both an existing clinical strategy and a challenge in advancing cancer immunotherapy.
- SIRPβ and SIRPγ represent next-generation therapeutic targets with tremendous potential.

## 1 Introduction

The three main members of the SIRP family, namely SIRPα, SIRPβ, and SIRPγ, share highly homologous extracellular domains but possess distinctly different signal transduction functions due to significant differences in their intracellular domain structures (1). SIRPα delivers inhibitory signals via immune receptor tyrosine-based inhibition motifs (ITIMs). In contrast, SIRPβ triggers activating signals by associating—via its transmembrane residue—with the ITAM-containing adaptor DAP12. SIRPγ has an extremely short cytoplasmic tail and was initially reported to function mainly in cell adhesion (2, 3). This “paired receptor” property enables the SIRP family to precisely regulate key immune processes such as phagocytosis, cell migration, inflammatory response, and T cell activation. In recent years, with the in-depth research in fields like tumor immunity, autoimmune diseases, and neurodegenerative diseases, the importance of the SIRP-CD47 axis has become increasingly prominent (4–7). In the tumor microenvironment, cancer cells highly expressing CD47 bind to SIRPα on macrophages and transmit a “don’t eat me” signal to achieve immune escape, making this pathway a hot target for cancer immunotherapy (8). Meanwhile, the activating signals mediated by SIRPβ have been proven to play a key role in inflammatory diseases and abnormal bone metabolism (9). In addition, in autoimmune diseases such as multiple sclerosis and type I diabetes, the dysregulated expression of SIRPγ on T cells is closely related to the occurrence and development of the diseases (10). The relationship between the above three proteins and CD47 is shown in Graphical Abstract. However, there is currently a lack of systematic comparison and summary of the three SIRP family members, and their expression regulation, signal crosstalk, and functional balance under different physiological and pathological conditions still need in-depth elaboration. Therefore, it is important to clearly describe the molecular structure, expression patterns, and signaling mechanisms of the main SIRP proteins (SIRPα, SIRPβ, and SIRPγ), as well as their interactions with ligands such as CD47. Based on this understanding, this review explains how these proteins function in immune balance and in major diseases including cancer, autoimmune, infectious, and neurodegenerative disorders. Finally, we discuss the opportunities and challenges of developing therapeutic antibodies that target the SIRP family.

## 2 Overview of the SIRP family

**Figure 1:**
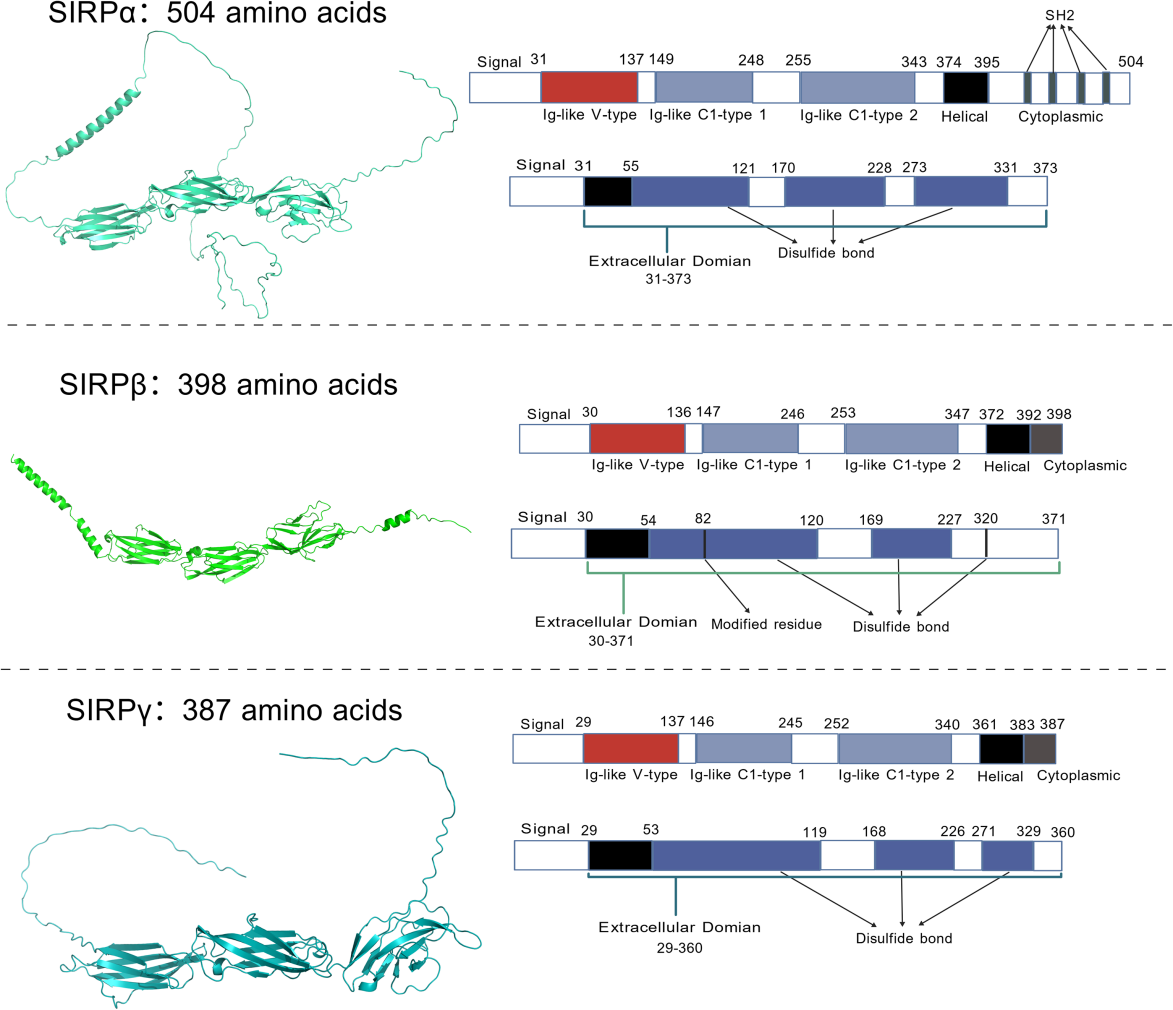
Schematic diagram of the SIRP protein structure and its intracellular and extracellular amino acid regions. The intracellular domain comprises a signal transduction region, an Ig-like V region, and Ig-like C1-type 1, C1-type 2, transmembrane regions (helical structures), and a cytoplasmic domain. The cytoplasmic domain of SIRPα contains four short spacer regions for SH2 binding. The cytoplasmic domain of SIRPβ is compact, consisting of only six amino acids, and lacks a phosphatase-binding signal motif. The cytoplasmic domain of SIRPγ is extremely short, comprising just four amino acids, with no known signal recruitment motifs identified. All extracellular domains contain disulfide bonds.

SIRPs are primarily expressed in myeloid cells and lymphocytes (11). They participate in various immune processes through interactions with their ligand CD47 (12). The first identified member of the SIRP family is rat protein tyrosine phosphatase SIRPα, also known as non-receptor substrate 1 (PTPNS1; alternatively designated as SHPS1, CD172A, and P84) (13, 14). The extracellular segment of SIRPα contains three immunoglobulin (Ig)-like superdomains (one V-set domain and two C1-set domains) and exhibits the highest binding affinity for CD47 (15). Its intracellular segment harbors a canonical immunoreceptor tyrosine-based inhibition motif (ITIM), which serves as the core of its “regulatory” function. Upon tyrosine phosphorylation, ITIM can recruit phosphatases such as SHP-1 and SHP-2, thereby transducing inhibitory signals (16). Following the discovery of SIRPα, researchers identified SIRPβ, a protein with structural similarity but opposing function. The extracellular domain of SIRPβ exhibits high homology with SIRPα. In contrast, no detectable binding between CD47 and SIRPβ has been identified, and its physiological significance remains unclear. To date, no natural high-affinity physiological ligand for SIRPβ has been identified (17). A key distinction in the intracellular segment from SIRPα is the absence of the ITIM motif; instead, it contains a positively charged residue that enables binding to adaptor proteins carrying immunoreceptor tyrosine-based activation motif (ITAM), such as DAP12 (13). The third well-characterized major subtype is SIRPγ, which can be regarded as a “streamlined version” of SIRPα in terms of structure (5). The extracellular domain exhibits high homology with the V domain of SIRPα, but its binding affinity for CD47 is relatively weak (17, 18). The differences between the protein structure and gene sequence of SIRP are shown in **Figure 1**.

All members of the SIRP family are type I transmembrane glycoproteins, with CD47 serving as their primary core ligand. CD47 is widely expressed on nearly all healthy “self” cells. Its binding to the SIRP family transmits a fundamental “self”-recognition signal (19). The most classic function is presenting a “don’t eat me” signal to innate immune cells (*e.g.*, macrophages). When SIRPα on macrophages binds to CD47 on healthy cells, it triggers an inhibitory signal mediated by SHP phosphatases, which strongly suppresses the phagocytic activity of macrophages, thereby protecting healthy cells from clearance. For adaptive immune cells (*e.g.*, T cells), it delivers a “don’t overactivate” signal (20). T cells themselves also express CD47 and SIRPγ (21). Notably, although CD47 and SIRPγ can interact, this study suggests that under acute activation conditions, the SIRPγ/CD47 interaction has limited effects on T cell proliferation and IFN-γ secretion. However, under chronic stimulation or sustained activation states, blocking SIRPγ/CD47 significantly inhibits IFN-γ secretion and affects T cell survival. This indicates that the SIRPγ/CD47 axis may exert differential regulatory roles at distinct stages of T cell activation, rather than functioning solely as a co-inhibitory signaling molecule (22).

## 3 Molecular structure and ligand binding

### 3.1 Structural characteristics of the SIRP family

The extracellular region of SIRP family members consists of three immunoglobulin superfamily (IgSF) domains: one membrane-distal V-set domain (D1) and two membrane-proximal C1-set domains (D2 and D3). The D1 domain serves as the key region responsible for binding to CD47, while the D2 and D3 domains may play a role in stabilizing the molecular structure (23, 24). Notably, SIRP molecules have alternative splicing variants, and some of these variants contain only the D1 domain. These D1-only variants may exist in a soluble form and regulate the availability of SIRP-CD47 interactions (24).

SIRPγ exists primarily as a monomer. SIRPβ forms homodimers via disulfide bonds, which likely influence its ligand binding and signaling properties (17, 25). While SIRPα is generally considered a monomer in solution, evidence suggests it can undergo cis dimerization at the cell membrane, potentially modulating its interaction with CD47 (24). This difference in oligomerization states may affect their ligand-binding modes and signal transduction properties. At the genetic level, genes of the SIRP family exhibit significant polymorphism, particularly in the ligand-binding region. Such genetic variations may influence an individual’s susceptibility to diseases, such as autoimmune diseases and infections (26).

### 3.2 Interactions of SIRPα, SIRPγ with CD47, and SIRPβ with DAP-12

CD47, the primary ligand of the SIRP family, is a widely expressed cell surface protein with a five-transmembrane structure, and its extracellular region contains a single IgV-like domain (27). CD47 is also referred to as integrin-associated protein (IAP) due to its ability to interact with and regulate the function of various integrins (28). Brooke G et al. employed surface plasmon resonance (SPR) technology to analyze the binding characteristics of soluble recombinant proteins (5). Biotin-labeled SIRP proteins were immobilized onto sensor chips, and monomeric CD47-CD4 (rat fusion protein) at varying concentrations was injected at 37 °C. Binding parameters were calculated through equilibrium binding and kinetic analysis, comparing the affinity differences between SIRPα and SIRPγ for binding CD47. The study revealed that SIRPα exhibits relatively high binding affinity for CD47 (Kd≈2μM), whereas SIRPγ demonstrates relatively low binding affinity (Kd≈23μM). The SIRPα-CD47 affinity is approximately 10 times that of SIRPγ-CD47 (5). SIRPα is preferentially activated under conditions of high CD47 expression, while SIRPγ may exert its effects when CD47 expression levels are relatively high. The expression level of CD47 is upregulated following T cell activation, which may influence its tendency to interact with different members of the SIRP family (29).

**Figure 2:**
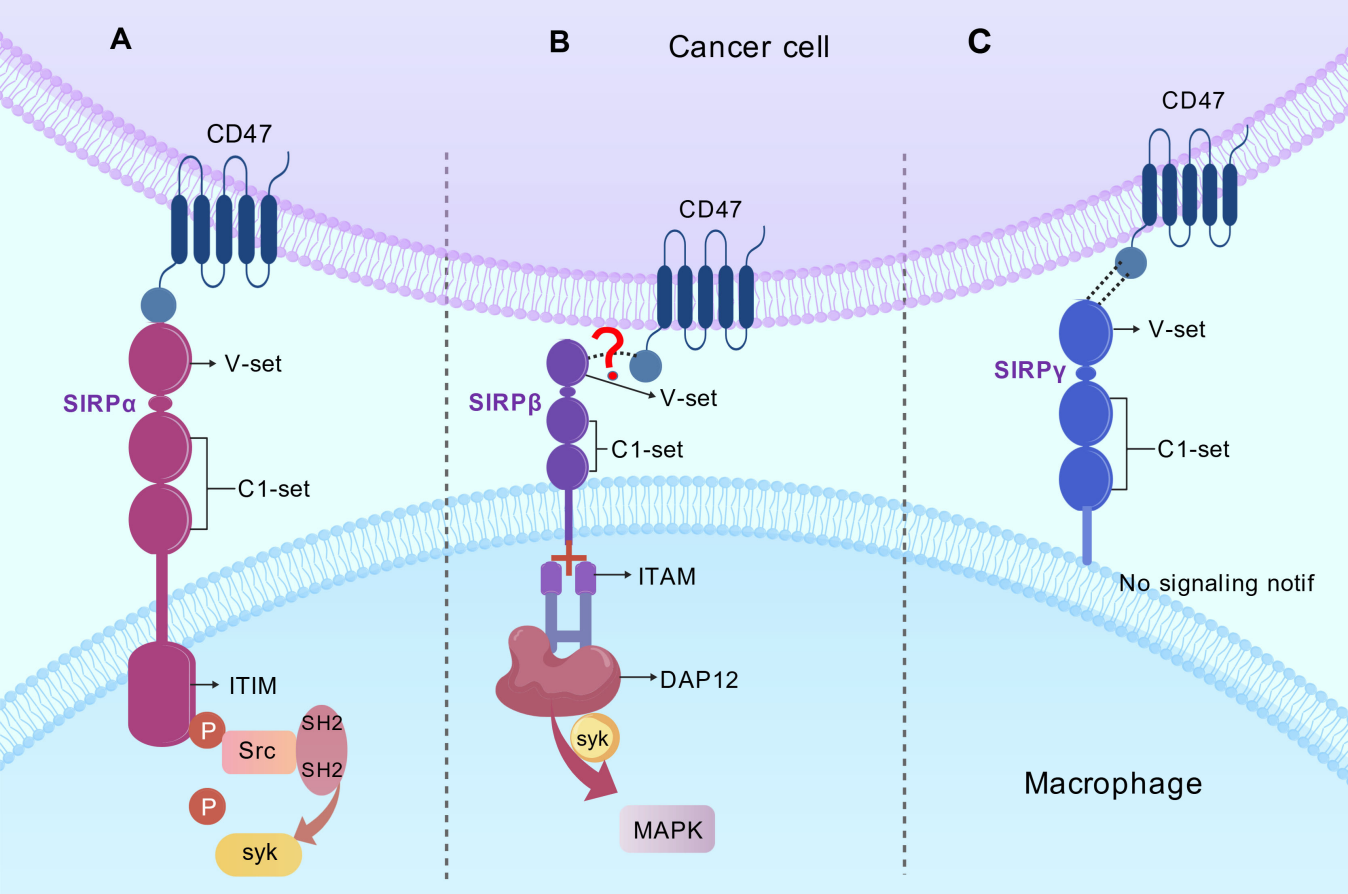
Structural distinctions within the SIRP family. SIRPα **(A)** possesses an architecture composed of one V-set domain and two C1-set domains. SIRPβ **(B)** exhibits high structural homology with SIRPα. SIRPβ interacts with the adaptor protein DAP12, which contains an ITAM motif, through positively charged amino acids in its transmembrane domain. SIRPγ **(C)** can be regarded as a “streamlined version” of SIRPα, typically described as a single transmembrane protein with an extremely short cytoplasmic tail.

DAP-12 exists as a homodimer, with each monomer containing one ITAM motif. Tyrosine phosphorylation of the two tyrosines within the ITAM leads to its association with SYK—a cytosolic tyrosine kinase that is crucial for antigen receptor signal transduction (17, 30). The binding between SIRPβ and DAP-12 is an ionic interaction between individual amino acids with opposite charges in their transmembrane domains (31, 32). SIRPβ appears to exert an opposite effect on cell activation compared to SIRPα; thus, it can be hypothesized that there may be a cooperative relationship between SIRPα and SIRPβ in signal regulation. These structural distinctions within the SIRP family were shown in **Figure 2**.

## 4 Signal transduction mechanisms

### 4.1 Inhibitory signaling of SIRPα

When SIRPα binds CD47, Src-family kinases phosphorylate tyrosines in SIRPα’s ITIMs. These phospho-ITIMs recruit SH2-domain phosphatases SHP1 and SHP2, which then dephosphorylate downstream targets to suppress activation (33–35). These phosphatases then dephosphorylate downstream signaling molecules, thereby inhibiting various cellular activities (36). In macrophages, the SIRPα-CD47 interaction provides a “don’t eat me” signal, preventing the phagocytosis of healthy self-cells that express CD47. This mechanism is particularly crucial in erythrocyte clearance—erythrocytes deficient in CD47 are rapidly cleared *in vivo* (37). Additionally, SIRPα signaling inhibits the production of proinflammatory cytokines (*e.g.*, TNF-α, IL-12) by myeloid cells and reduces the migration of neutrophils in collagen (38).

### 4.2 Activating signaling of SIRPβ

In contrast to SIRPα, SIRPβ transduces activating signals. It interacts with the ITAM-containing adaptor protein DAP12 via positively charged amino acids in its transmembrane region (39). When SIRPβ is cross-linked, the ITAM motif in DAP12 becomes phosphorylated, recruiting and activating SYK (spleen tyrosine kinase), which further activates the MAPK (mitogen-activated protein kinase) pathway and other downstream signaling cascades (17, 40). Studies have shown that cross-linking SIRPβ with specific antibodies can enhance the phagocytic capacity of macrophages and the migratory ability of neutrophils. However, the natural ligand of SIRPβ has not yet been identified, which limits the understanding of its *in vivo* functions (25, 41).

### 4.3 SIRPγ does not directly transduce intracellular signals

SIRPγ is a member of the SIRP family with a unique structure. It lacks intrinsic signal transduction capability and primarily functions as an adaptor or regulatory molecule (5). SIRPγ exhibits weak binding affinity for CD47. This receptor was initially designated SIRPβ2 but is now uniformly named SIRPγ. Its cytoplasmic domain is extremely short (comprising only 4 amino acids), lacks known signal transduction motifs, and does not bind to DAP12. thus, it has traditionally been regarded as a decoy receptor (5, 18, 42). Nevertheless, studies have demonstrated that SIRPγ is mainly expressed on the surface of T cells, while its ligand CD47 is expressed on antigen-presenting cells (*e.g.*, dendritic cells). The interaction between SIRPγ and CD47 is believed to provide a costimulatory signal for T cell receptor (TCR) signaling, enhancing T cell activation. However, SIRPγ itself does not transmit signals; instead, it may indirectly regulate signaling by forming complexes with other transmembrane proteins (*e.g.*, integrins) (22). Moreover, SIRPγ can competitively bind to CD47 with SIRPα. Since SIRPγ lacks an inhibitory cytoplasmic segment, its binding to CD47 does not transduce the “don’t eat me” signal, thereby essentially attenuating the inhibitory function of SIRPα. This mechanism enables the fine-tuning of the intensity of immune responses in specific microenvironments (22, 43, 44). The above three signal transduction mechanisms are summarized in the table below (**Table 1**).

**Table 1:** Comparison of structural and functional features of the SIRP family.

Feature	Key structural features	Extracellular domains	Ligand and binding characteristics	Signal transduction pathway	Key features	Representative references
SIRPα (inhibitory)	The cytoplasmic region contains ITIM	Three Ig-like domains (V-type + 2 C1-type)	High affinity binding to CD47	Phosphorylated → of ITIM recruits SHP1/SHP2 → to dephosphorylate downstream molecules	Transmit the “don’t eat me” signal in macrophages to prevent phagocytosis of healthy own cells. Inhibition of pro-inflammatory cytokines produced by myeloid cells. Reduces neutrophil migration.	(13, 33–37)
SIRPβ (activation)	The transmembrane region contains positively charged amino acids and binds to DAP12	Three Ig-like domains (V-type + 2 C1-type)	May bind to CD47 low affinity; The natural ligand is unknown	The ITAM phosphorylation → of DAP12 recruits downstream molecules such as activating SYK → activating the MAPK pathway	Enhance macrophage phagocytosis. Enhance neutrophil migration.	(17, 39, 40)
SIRPγ (No direct signal)	The cytoplasmic region is extremely short (4 amino acids), has no known signal, and does not bind to DAP12	Three Ig-like domains (V-type + 2 C1-type)	Binding to CD47, the affinity is between SIRPα and SIRPβ	Does not directly transduce signals; May act as a adapter or regulatory molecule	It is expressed on the surface of T cells, competitively binding to CD47, weakening the inhibitory function of SIRPα and finely regulating the intensity of immune response.	(5, 42–44)

## 5 Association of the SIRP family with diseases

### 5.1 SIRPα: a multifunctional regulator of the “don’t eat me” signal

SIRPα plays a pivotal role in both health and disease. Together with CD47, it acts as a negative regulator of phagocytosis in innate immune cells (45). SIRPα is primarily expressed in myeloid cells, macrophages, dendritic cells (DCs), natural killer (NK) cells, as well as monocytes in humans and other mammals (46), serving as a marker inhibitory receptor for myeloid cells. In cancer, animal xenotransplantation, neuroimmunity, infectious immunity, and the cardiovascular system (46), Blocking the SIRPα-CD47 axis using anti-SIRPα or anti-CD47 antibodies can mobilize multiple immune cells to enhance antitumor immunity. In humans or mice, host NK cells can recognize molecules lacking their own MHC molecules, while allogeneic transplant cells are phagocytosed by host cells due to the binding of heterologous CD47 on the graft to host SIRPα deficiency. Binding of CD47 on recipient monocytes to SIRPα on transplanted cells induces activation signals, leading to proliferation and differentiation of mouse monocytes (47). This process contributes to terminal graft rejection during allogeneic transplantation in animals, demonstrating the role of this blockade strategy as a critical regulatory mechanism (7, 46, 48).

**Figure 3:**
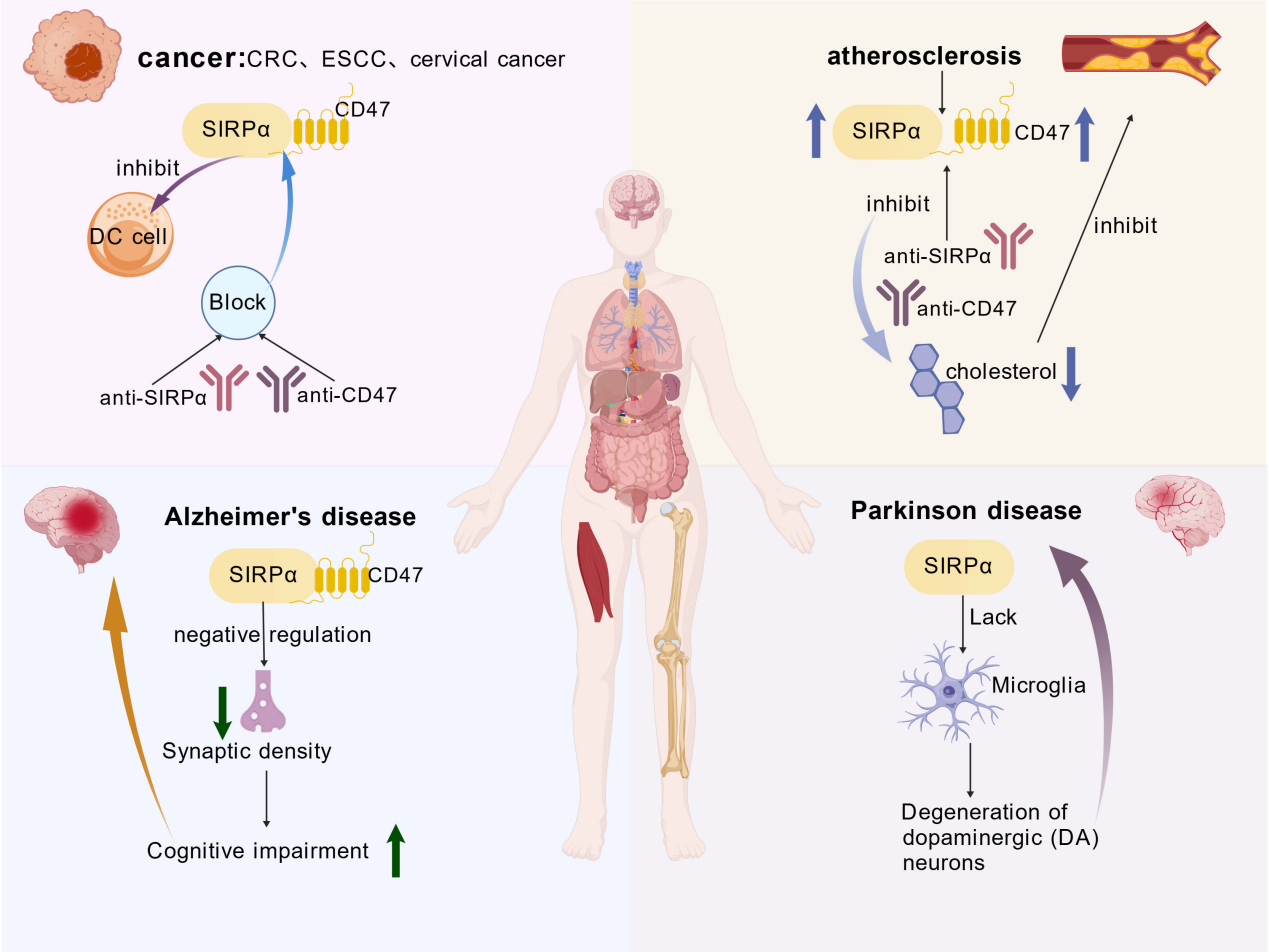
The function and impact of SIRPα in disease. These include cancer and atherosclerosis, Alzheimer’s disease, Parkinson’s disease, and so on. SIRPα binding to CD47 suppresses DC cell expression, blocking their interaction via antibody drugs achieves therapeutic effects against cancer. In atherosclerotic disease, SIRPα and CD47 expression is upregulated. Myeloid cell-specific SIRPα deficiency inhibits atherosclerotic lesion formation, while CD47 deficiency suppresses macrophage proliferation. Parkinson’s disease and Alzheimer’s disease have been discussed above.

The CD47/SIRPα pathway mediates cancer immune escape and immunotherapy, and plays a crucial role in cancers such as colorectal cancer(CRC), esophageal squamous cell carcinoma(ESCC), and cervical cancer (16, 49, 50). The role of the CD47/SIRPα interaction in providing cancer cells with a macrophage-targeted escape mechanism has been fully characterized. Studies have shown that drugs targeting CD47/SIRPα have been developed and exhibit preclinical activity; research on CD47/SIRPα-directed drugs based on existing data has demonstrated their safety and preliminary activity (51). In addition, the CD47/SIRPα interaction also exerts effects on apoptosis, proliferation, and migration of tumor cells (52, 53). Shi L et al. found that SIRPα deficiency, in turn, attenuates the inhibitory effect mediated by SIRPα, thereby increasing the pro-inflammatory polarization of macrophages and further exacerbating the pro-inflammatory response in mouse models of type 1 diabetes and peritonitis (18, 54). Furthermore, researchers have discovered that the SIRPα-CD47 interaction inhibits the clearance of apoptotic cells by macrophages and promotes the formation of plaque necrotic cores. Myeloid cell-specific SIRPα deletion suppresses the formation of atherosclerotic lesions, while CD47 deletion inhibits macrophage proliferation. These findings identify SIRPα as a potential target for atherosclerosis and highlight the importance of cell-specific CD47 inhibition as a future therapeutic strategy (55). Hana H et al. demonstrated that mice with experimental visceral leishmaniasis (VL) exhibit anemia and enhanced splenic hemophagocytosis, accompanied by decreased SIRPα expression. Serum soluble SIRPα can serve as a biomarker for hemophagocytosis and anemia in VL and other inflammatory diseases (56). Ding X et al. used experiments such as conditional knockout mice to show that microglia-specific deletion of SIRPα leads to a reduction in synaptic density. A decrease in microglial SIRPα expression was observed in human tissues with the progression of Alzheimer’s disease(AD) (57, 58). Wang J et al. evaluated the effect of SIRPα downregulation on the pathogenesis of Parkinson’s disease(PD) using cell culture and animal models. During aging or inflammatory challenge, the level of SIRPα in microglia decreases; downregulation of SIRPα unleashes the inflammatory response of microglia, thereby revealing the inhibitory effect of SIRPα on microglial activation (59). In summary, as a key immunomodulatory receptor, SIRPα maintains tissue homeostasis (*e.g.*, protecting red blood cells) by transmitting “don’t eat me” signals. However, its abnormal activation can also promote diseases (*e.g.*, cancer immune escape and atherosclerosis). Therefore, SIRPα is a potential target for the treatment of autoimmune diseases, cancer, and cardiovascular diseases. Its functional roles and impacts in disease are illustrated in **Figure 3**.

### 5.2 SIRPβ: unique activating immune receptor

SIRPβ is predominantly expressed in myeloid cells, such as neutrophils, monocytes, macrophages, and dendritic cells (32). Its expression levels and activation status may differ in specific inflammatory or tumor microenvironments. It is constitutively expressed on the cell membrane, and its expression is not significantly upregulated upon activation (60). In contrast to the function of SIRPα—where SIRPα inhibits migration—SIRPβ promotes migration, forming a bidirectional regulatory mechanism that finely modulates inflammatory responses (61). As an activating immune receptor, SIRPβ transmits signals through DAP12 to promote inflammatory cell migration and osteoclast differentiation (25).

**Figure 4:**
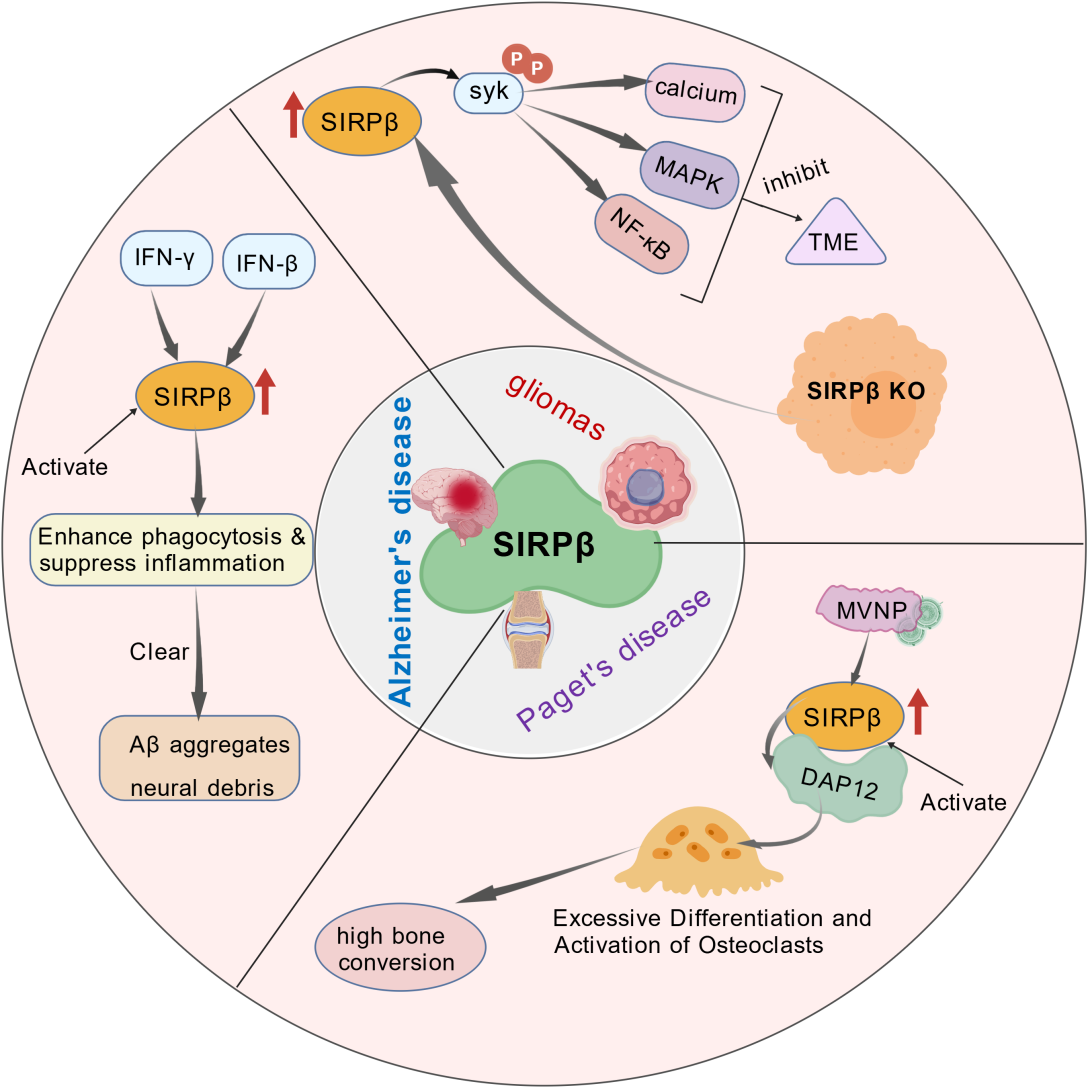
The function and impact of SIRPβ in disease. In Alzheimer’s disease, interferons (IFN-γ and IFN-β) produced in the inflammatory environment stimulate microglia, upregulating the transcription and expression of SIRPβ. By activating SIRPβ, phagocytosis is enhanced and inflammation is suppressed, thereby clearing Aβ and neurotoxic debris. In Paget’s disease, MVNP significantly upregulates SIRPβ expression. This enhances the interaction between SIRPβ and its signaling partner DAP12, leading to excessive osteoclast formation and hyperactive bone resorption. Increased SIRPβ expression in oligodendroglial cells leads to Syk phosphorylation, affecting calcium ion, MAPK, NF-κB, and other signaling pathways, thereby influencing the tumor immune microenvironment.

Hayashi A et al. found that SIRPβ binding promotes macrophage phagocytosis by inducing tyrosine phosphorylation of DAP12, SYK, and SLP-76, followed by activation of the MEK-MAPK-myosin light chain kinase cascade (41). Lahoud MH et al. confirmed the differential expression of SIRPα and SIRPβ molecules on mouse dendritic cells (DCs) via monoclonal antibody staining, which included a novel monoclonal antibody that recognizes SIRPβ. Cross-linking of SIRPβ on DCs led to a reduction in the phagocytosis of major Leishmania parasites, but had no effect on the phagocytosis of latex beads. This finding may indicate that the regulation of phagocytosis by SIRPβ is a ligand-dependent interaction (62). Geng R et al. identified significantly elevated SIRPβ expression in gliomas, which adversely affects the immune microenvironment and correlates poorly with patient survival. Glioma cells can activate macrophages via SIRPβ, subsequently reprogramming the tumor microenvironment (TME), suggesting SIRPβ as a promising therapeutic target for gliomas (9). Gaikwad S et al. found that SIRPβ expression is upregulated on microglia in amyloid precursor protein J20 (APP/J20) transgenic mice and patients with Alzheimer’s disease (AD), and it functions as a phagocytic receptor (63, 64). Sundaram K et al. found that measles virus nucleocapsid protein (MVNP) upregulates SIRPβ expression, promoting osteoclast differentiation in Paget’s disease of bone (PDB) (65). In summary, we conclude that SIRPβ enhances immune cell functions (such as phagocytosis and migration) under healthy conditions through DAP12-mediated activation signals, while its excessive activation in disease states contributes to pathological processes. Its functional roles and impacts in disease are illustrated in **Figure 4**.

### 5.3 SIRPγ: an adaptor or regulatory T cell adhesion receptor

SIRPγ is predominantly expressed on T lymphocytes; it is also expressed on T cells, CD56 natural killer (NK) cells, and all activated NK cells, presenting adaptive immune specificity (17, 22, 66). Compared with SIRPα, the relative orientation between the D1 domain and D2-D3 domains of SIRPγ is more flexible, which may affect its function. SIRPγ does not bind to signal adaptor proteins such as DAP12, nor does it possess obvious signal transduction function (1, 67). Although SIRPγ itself has no signal transduction function, its weak interaction with CD47 may regulate the activation threshold of T cells or the stability of intercellular contacts (68). Under chronic stimulation conditions, it positively regulates T cell effector functions (such as IFN-γ secretion). This action is independent of integrins and may play an auxiliary role in T cell adhesion to antigen-presenting cells (APCs). Under acute activation conditions, its function may be more oriented toward maintaining T cell activity during sustained immune responses (22, 69).

**Figure 5:**
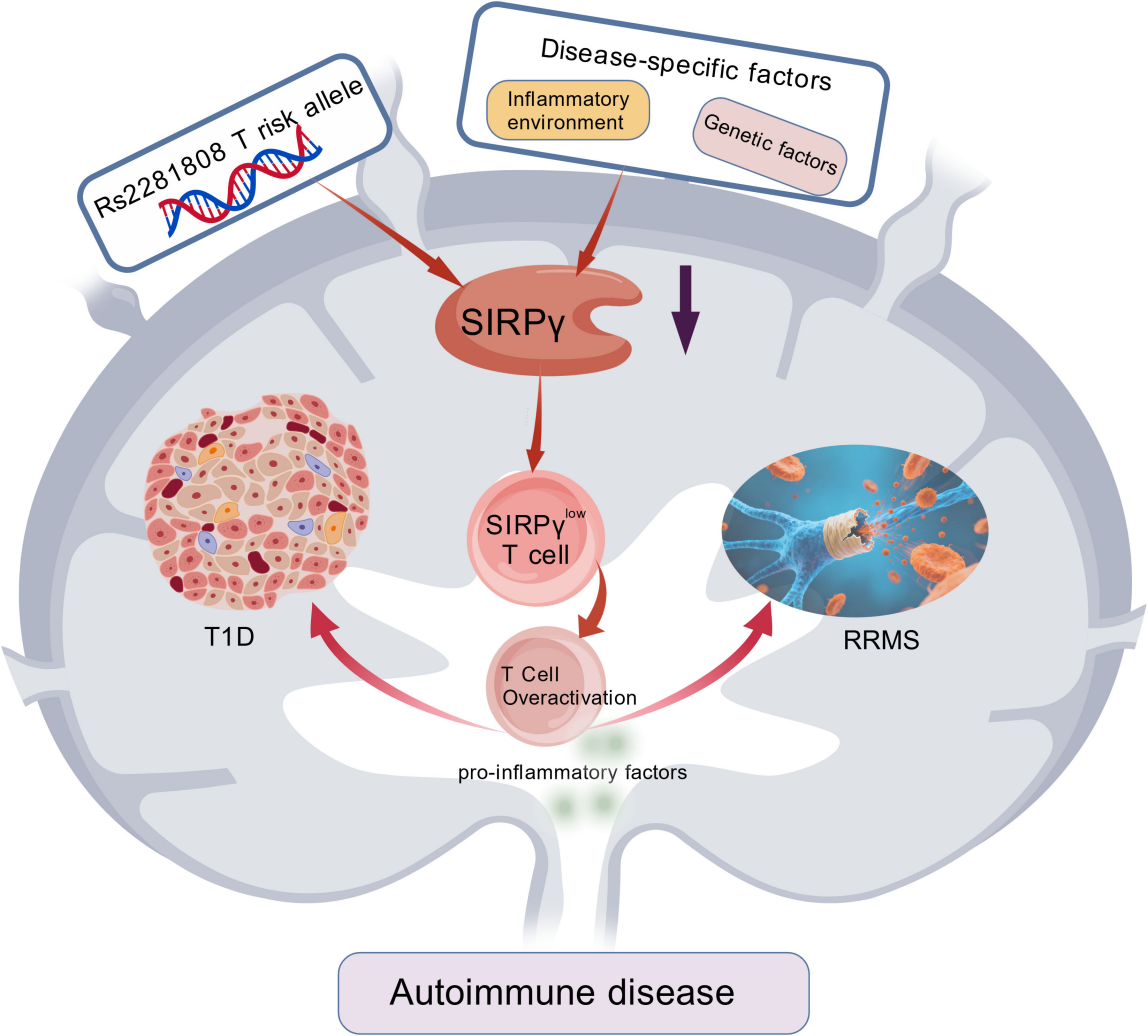
The function and impact of SIRPγ in disease. Reduced SIRPγ expression (resulting from both genetic factors and disease environments) releases inhibition on T cells, generating hyperactivated SIRPγ^low^ T cells. These cells exhibit enhanced effector functions and pathogenicity, ultimately driving target organ inflammation and damage in autoimmune diseases such as RRMS and T1D.

Visser N et al. found that SIRPβ2 is a novel positive regulator of innate anti-cancer immunity and a potential co-stimulatory target for innate immunotherapy. In addition, ectopic expression of SIRPβ2 stimulates macrophage adhesion, differentiation, and cancer cell phagocytosis, as well as enhances macrophage-mediated T cell receptor-specific T cell activation (3). Besides, SIRPγ determines the cancer stem-like cell (CSLC) properties and immune escape ability in a small subset of lung adenocarcinoma (LUAD) cancer cells. Targeting SIRPγ via SIRPγ gene knockdown or SIRPγ-neutralizing antibodies can inhibit the CSLC phenotype and induce phagocytosis, thereby suppressing tumor growth *in vivo* (70). Nettleship JE et al. obtained SIRP crystals by forming complexes between the protein and the Fab fragment of the anti-SIRP antibody OX117; this method was proven effective for crystallizing human SIRPγ and subsequently obtaining antigen complexes (71). Sinha S et al. found that the expression of SIRPγ on T cells in patients with relapsing-remitting multiple sclerosis (RRMS) and type 1 diabetes (T1D) is significantly lower than that in healthy individuals, and this difference is not fully explained by genetic variations—suggesting that disease-specific factors may also regulate SIRPγ expression. The increased frequency of SIRPγ-low T cells is associated with pro-inflammatory molecules in T cells, indicating that the dysregulation of SIRPγ expression on T cells may play a key role in immune dysregulation in autoimmune diseases (22, 72, 73). The above results indicate that under healthy conditions, SIRPγ maintains immune homeostasis by promoting T cell adhesion, migration, and activation; in autoimmune diseases (such as multiple sclerosis and diabetes), its downregulated expression leads to excessive T cell activation and aggravated inflammation. Meanwhile, during chronic immune responses, SIRPγ enhances T cell effector function through interaction with CD47. However, its specific antibodies may avoid interfering with T cell function and thus hold therapeutic potential. Its functional roles and impacts in disease are illustrated in **Figure 5**. [Created with BioGDP.com (74)].

## 6 The SIRP family as therapeutic targets for disease resistance

### 6.1 Current status of drug research targeting SIRP

Currently, drug development targeting SIRP primarily focuses on SIRPα, aiming to unblock its interaction with CD47 and thereby release the suppression of phagocytes such as macrophages. This approach seeks to activate the innate immune system to eliminate cancer cells or diseased cells. Qu T et al. (75) reviewed the development, safety, and efficacy of drugs targeting the CD47/SIRPα axis in preclinical and clinical studies. Three types of drugs targeting this axis have been developed: anti-CD47 antibodies, SIRPα-Fc fusion proteins, and anti-SIRPα antibodies.

First, antibodies targeting CD47 function by blocking the CD47/SIRPα interaction, thereby restoring macrophage phagocytic activity. Some also induce tumor cell apoptosis and adaptive immunity (76). Representative agents such as Magrolimab (Hu5F9-G4) (77, 78) have entered Phase III clinical trials. When combined with rituximab and azacitidine, it demonstrated efficacy in non-Hodgkin lymphoma and acute myeloid leukemia (AML), but toxicity such as anemia necessitates a low-dose initiation strategy (79). Ligufalimab (AK117) lacks hemolytic activity and requires no dose reduction (80), Lemzoparlimab (TJC4) exhibits low erythrocyte toxicity (81), AO176 directly kills tumor cells, CC-90002 and SGN-CD47M faced trial discontinuations due to efficacy or safety concerns (75).

**Figure 6:**
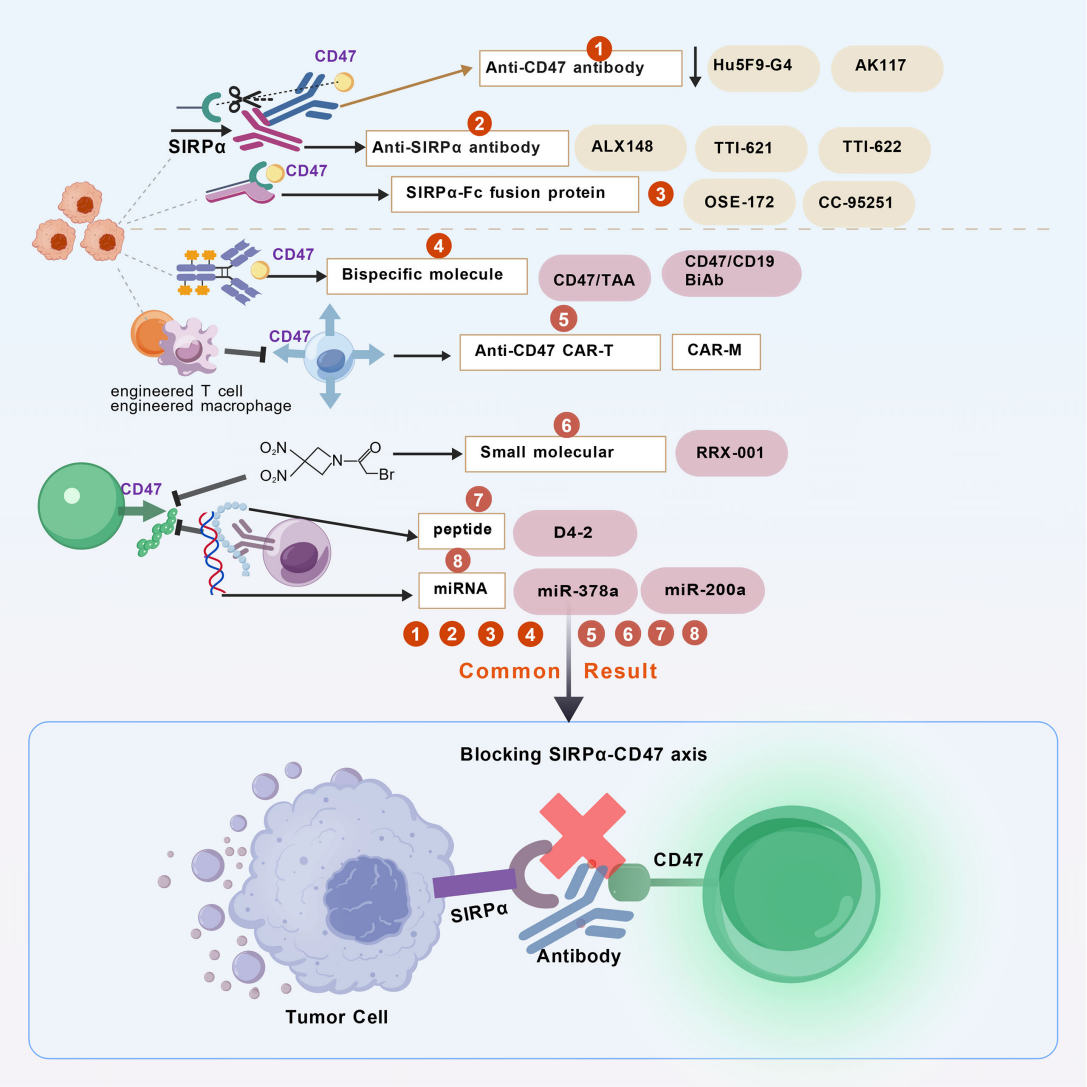
Mechanism of drug action. The eight drugs summarized above all achieve the same outcome by blocking the interaction between SIRPα and CD47, thereby restoring macrophage phagocytic activity and eliminating tumor cells both *in vivo* and *in vitro*.

Second, the SIRPα-Fc fusion protein blocks inhibitory signals by binding to CD47 on tumor cells, thereby enhancing phagocytic activity. Wild-type SIRPα exhibits low affinity for CD47, necessitating affinity enhancement through modification (82). Representative drugs such as Evorpacept (ALX148) demonstrate significantly improved affinity and show efficacy in combination with multiple agents for lymphoma and solid tumors (83), TTI-621 and TTI-622, based on wild-type SIRPα, may cause transient anemia (75), IMM01 does not bind to red blood cells and exhibits synergistic effects when combined with rituximab (84). Third, antibodies targeting SIRPα (85) exhibit weak phagocytic activity when used alone but show significantly enhanced activity when combined with mictropic antibodies. Concerns include Fc effector toxicity, cross-reactivity with SIRPγ, antibody internalization, and coverage of SIRPα subtypes. Representative drugs such as OSE-172, CC-95251, and FSI-189 are mostly in early clinical stages (75). The CD47/SIRPα axis is a critical target in tumor immunotherapy, with antibodies, fusion proteins, and bispecific molecules targeting this axis demonstrating promising efficacy in hematologic malignancies. In addition, bispecific molecules (85, 86), engineered T cells and macrophages (87), small molecules (88), peptides (89), and miRNAs (90). Examples include NI-1701, IMM0306 (85, 86), CD47-CAR-T cells, CAR-M (chimeric antigen receptor macrophages) (87), RRx-001 (88), D4-2, PKHB1 (89), miR-378a, miR-200a, and miR-708 (90). The drug targets, mechanisms, trial phases, key adverse events, and statuses listed above are shown in **Table 2**. The mechanism of action of the drug is shown in **Figure 6**. Unlike therapies targeting SIRPα, no drugs targeting SIRPβ have yet entered clinical development. Given the central role of innate immunity in the tumor microenvironment, combination therapies integrating SIRPβ agonists with PD-1/PD-L1 inhibitors, targeted antibodies, and chemotherapy may emerge as a primary research direction. In publicly reported studies, drug development targeting SIRPγ remains in an early and relatively limited stage. The field of SIRPγ-targeted therapy represents a “blue ocean,” with no molecules currently entering clinical trials. Related research remains exploratory and preclinical.

**Table 2:** Representative drug targets and phase summary.

Drug name	Target/mechanism	Mode of action	Experimental phase	Key AEs (Adverse Reactions)	State	References
Magrolimab (Hu5F9-G4)	Anti-CD47 monoclonal antibody, blocking CD47/SIRPα	IgG4 humanized monoclonal antibody	in multiple phase III trials	Anemia, thrombocytopenia, transient hematopoietic toxicity (low dose initiation required)	Several trials are ongoing	(77, 78)
Ligufalimab (AK117)	Anti-CD47 monoclonal antibody, blocking CD47/SIRPα	IgG4 humanized monoclonal antibody	Phase I/II	There was no significant anemia and no dose-limiting toxicity was observed	Recruiting	(75, 80)
Lemzoparlimab (TJC4)	Anti-CD47 monoclonal antibody, blocking CD47/SIRPα	IgG4 humanized monoclonal antibody	Phase III	Low cytotoxicity	Recruiting	(75, 80, 81)
AO-176	Anti-CD47 monoclonal antibody, blocking CD47/SIRPα	IgG2 humanized monoclonal antibody	Phase I/II	Anemia was mild, and no anemia was observed in the monkey model; DLT occurs at 20 mg/kg	Completed	(75, 80)
CC-90002	Anti-CD47 monoclonal antibody, blocking CD47/SIRPα	IgG4 humanized monoclonal antibody	Phase I (Terminated)	Anemia (50%) and thrombocytopenia (33%) have poor efficacy and safety	Terminated	(75, 80)
SGN-CD47M	CD47 targeting precursor drug conjugates	Prodrug conjugates (PDCs)	Phase I (Terminated)	It was not reported that the trial was terminated due to project prioritization adjustments	Terminated	(75)
Evorpacept (ALX148)	SIRPα-Fc fusion protein, mutant high affinity binding to CD47	SIRPα mutant-Fc (IgG1)	Phase II/III	Mild anemia, tolerated in combination with chemotherapy	Several trials are ongoing	(75)
TTI-621	SIRPα-Fc fusion protein (wild-type V2D1-IgG1)	SIRPα-Fc fusion protein	Phase I/II	Transient anemia and thrombocytopenia, recovery within 7 days	Recruiting	(75, 80)
TTI-622	SIRPα-Fc fusion protein (wild-type V2D1-IgG4)	SIRPα-Fc fusion protein	Phase I/II	It is well tolerated to 18 mg/kg for transient hematopoietic toxicity	Recruiting	(75, 80)
IMM01	SIRP α-Fc fusion protein (V2D1 N80A mutation)	SIRPα-Fc fusion protein	Phase I/II	Transient platelet drop, recovery in 24 to 48 hours	Recruiting	(75, 84)
OSE-172 (BI 765063)	Anti-SIRP α monoclonal antibody	IgG4 humanized monoclonal antibody	Phase I/II	Not specified, trials are ongoing	Recruiting	(75, 91)
CC-95251	Anti-SIRP α monoclonal antibody	Humanized monoclonal antibody	Phase I	Not specified, trials are ongoing	Recruiting	(75)
FSI-189	Anti-SIRP α monoclonal antibody	IgG1 humanization (no effective function)	Phase I	Not specified, trials are ongoing	Recruitment has not begun	(75)
NI-1701 (TG-1801)	CD47 × CD19 bispecific antibodies	IgG1 (KIH structure)	Phase I	Avoid binding to normal cells and reduce the “antigen groove” effect	Early clinical trials	(75, 85)
IMM0306	CD47 × CD20 bispecific antibody (SIRPα-Fc fusion)	SIRPα-Fc is fused with rituximab	Phase I	Not specified, trials are ongoing	In progress	(75, 86)
CD47-CAR-T cell	CD47 is the target CAR-T	CAR-T cell therapy	Preclinical stage	Underlying CRS (cytokine release syndrome) and cytotoxicity	Preclinical research	(75, 87)
CAR-M (Chimeric antigen receptor macrophages)	Engineered macrophages targeting tumor-associated antigens	Engineered macrophages	Preclinical stage	Underlying CRS with macrophage-mediated inflammatory responses	Preclinical research	(75, 87)
RRx-001	Down-regulated small molecules expressed by CD47 and SIRPα	Small molecule drugs	Phase III(SCLC)	Not specified	In progress	(75, 88)
D4-2	Macrocyclic peptide inhibitors targeting mouse SIRPα	Macrocyclic peptides	Preclinical stage	Not specified	Preclinical research	(75, 89)
PKHB1	TSP-1 derives CD47 agonist peptide, which induces cell death	Peptide agonists	Preclinical stage	Not specified	Preclinical research	(75, 89)
miR-378a, miR-200a, miR-708	Downregulate CD47 or SIRPα expressed microRNAs	microRNA therapy	Preclinical stage	Not specified	Preclinical research	(75, 90)

### 6.2 Antibody drugs indirectly inhibit the SIRPα-CD47 axis

Extensive research indicates that CD47 is highly expressed in various hematologic malignancies and solid tumors, where it binds to SIRPα on tumor-associated macrophages (92). Its overexpression is significantly associated with poor patient prognosis. Therefore, blocking the SIRPα-CD47 interaction to reactivate the antitumor function of macrophages presents a novel opportunity for cancer immunotherapy. These drugs target CD47 by indirectly blocking its interaction with SIRPα (93). For example, the representative anti-CD47 drug Hu5F9-G4 is a humanized IgG4 antibody. It specifically binds to the extracellular domain (CD47-ECD) of the CD47 molecule, forming an antibody-antigen complex (94). The heavy chain variable region (VH) and light chain variable region (VL) of this antibody cover approximately 365 Å² and 310 Å² of the CD47-ECD surface area, respectively, blocking the interaction between CD47 and its natural ligand SIRPα through a steric hindrance effect (94). Structure alignment analysis conducted by Huang B et al. using Discovery Studio 2019 revealed that the binding epitope of Hu5F9-G4 on CD47 highly overlaps with the SIRPα binding region, primarily involving key domains on the CD47 surface such as the BC loop and FG loop. The CDR loops of Hu5F9-G4 form 11 hydrogen bonds with the surface of the CD47 extracellular domain (ECD) (94).

## 7 Conclusion and future perspectives

Therapeutics targeting the SIRP-CD47 axis (particularly anti-CD47/SIRPα antibodies) have demonstrated potential in cancer immunotherapy by promoting macrophage phagocytosis, positioning them as promising therapeutic targets. As the star molecule of the family, SIRPα remains a focal point of current research. Previous studies have revealed significant advances in understanding SIRPα’s immunoregulatory mechanisms and its association with various diseases. The extracellular domains of SIRPβ and SIRPα, particularly the first IgV domain that binds CD47, exhibit extremely high sequence and structural homology. Despite this similarity, SIRPβ possesses several unique amino acid residues within the critical CD47-binding loop region. These subtle differences prevent SIRPβ from binding CD47 with high affinity. Consequently, identifying its ligand cannot be advanced simply by studying CD47 interactions. The natural ligand for SIRPβ remains unidentified, stemming from its structural similarity to SIRPα yet distinct functionality, coupled with the low affinity, multivalent nature, and potential complexity of its ligand interactions. Although capable of transmitting activation signals via DAP12, identifying SIRPβ’s natural ligand and its specific role in additional diseases presents significant challenges. The precise mechanism by which SIRPγ modulates T cell function through collaboration with other receptors, such as integrins, remains unclear. Drug development targeting SIRPβ and SIRPγ also presents substantial challenges. Given the distinct yet interdependent roles of SIRP family members in immune regulation, their in-depth investigation will provide novel targets and strategies for immunotherapy across multiple diseases.
